# Being as Normal as Possible: How Young People Ages 16–25 Years Evaluate the Risks and Benefits of Treatment for Inflammatory Arthritis

**DOI:** 10.1002/acr.22832

**Published:** 2016-07-28

**Authors:** Ruth I. Hart, Janet E. McDonagh, Ben Thompson, Helen E. Foster, Lesley Kay, Andrea Myers, Tim Rapley

**Affiliations:** ^1^ University of YorkYorkUK; ^2^ University of ManchesterManchesterUK; ^3^ Newcastle Hospitals NHS Foundation TrustNewcastle‐upon‐TyneUK; ^4^ Newcastle Hospitals NHS Foundation Trust and Newcastle UniversityNewcastle‐upon‐TyneUK; ^5^ Northumbria Healthcare NHS Foundation TrustNorth ShieldsUK; ^6^ Newcastle UniversityNewcastle‐upon‐TyneUK

## Abstract

**Objective:**

To explore how young people (ages 16–25 years) with inflammatory arthritis evaluate the risks and benefits of treatment, particularly treatment with biologic therapies.

**Methods:**

This qualitative study involved in‐depth interviews (n = 44) with young people, trusted others (e.g., parents), and health professionals; audio‐recordings (n = 4) of biologic therapy–related consultations; and focus groups (n = 4). Analysis used techniques from grounded theory (open and focused coding, constant comparison, memoing, and mapping).

**Results:**

Young people aspired to live what they perceived as a “normal” life. They saw treatment as presenting both an opportunity for and a threat to achieving this. Treatment changes were therefore subject to complex and ongoing evaluation, covering administration, associated restrictions, anticipated effects, and side effects. Information sources included expert opinion (of professionals and other patients) and personal experience. Previous treatments provided important reference points. Faced with uncertain outcomes, young people made provisional decisions. Both trusted others and health professionals expressed concern that young people were too focused on short‐term outcomes.

**Conclusion:**

Young people value treatment that helps them to live a “normal” life. There is more to this than controlling disease. The emotional, social, and vocational consequences of treatment can be profound and lasting: opportunities to discuss the effects of treatment should be provided early and regularly. While making every effort to ensure understanding of the long‐term clinical consequences of taking or not taking medication, the wider impact of treatment should not be dismissed. Only through understanding young people's values, preferences, and concerns can a sustainable balance between disease control and treatment burden be achieved.

## INTRODUCTION

There is increasing consensus that patient preferences are both important and unpredictable. What patients want is not necessarily what doctors think they want 1. Within rheumatology, the body of studies examining patient treatment preferences is growing. However, research to date has focused on older adults with rheumatoid arthritis 2, 3, 4, 5.

Box 1 **Significance & Innovations**

- Prior research on patient treatment preferences has focused on older adults with rheumatoid arthritis. Young people with inflammatory arthritis have different circumstances and concerns.
- Young people see treatment as presenting both an opportunity and a threat to their desire to lead a normal life. They describe a wide range of consequences (physical, emotional, social, and vocational) arising from their treatment regimens.
- In evaluating treatment changes, young people take this wide range of outcomes into account; where outcomes are uncertain they consider decisions to be provisional.
- Young people need active encouragement to discuss their treatment concerns and difficulties with the care team, so that a sustainable balance between disease control and treatment burden can be achieved.

Like older adults, young people with inflammatory arthritis (IA) can have severe disease warranting aggressive treatment, including biologic agents 6. In other respects, however, they are different. Clinical differences set young people with juvenile idiopathic arthritis (JIA) apart from adult patients. Developmental differences further distinguish them (and young people with other forms of IA) from adult patients, who bring fully matured brains to bear on their decision‐making 7. The social context in which young people make decisions about managing their disease also differs in important ways from that of older patients, and may have a profound effect on decision‐making 8.

Choice is exercised within and outside the clinic. Patients make decisions about treatment options in the context of consultations, and then, on a routine basis, whether and how to enact their agreed upon regimen. Evidence of the link between patient preferences and adherence is increasingly convincing: where treatment decisions align with patient preferences, clinical outcomes are better 1, 9, 10. Treatment choices may also promote (or impede) the achievement of key developmental milestones, such as establishing a career and a family. So the consequences of treatment decisions made early in life may affect both short‐ and longer‐term health, intrude into other domains, and extend through the life course.

There are a variety of reasons that young people's treatment preferences might differ from those of older adults. Understanding how they inform treatment choices matters, due to the profound and lasting impact that such decisions have. Our study therefore explored how young people evaluate the risks and benefits of treatments, in particular biologic therapies. It considered their priorities and concerns and the challenges treatment presented. Other aspects of the work (relating to the influence of “trusted others” on decision‐making) have been reported elsewhere 11.

## PATIENTS AND METHODS

We report findings from a qualitative study conducted in England, 2012–2014. The study explored decision‐making about biologic therapies by young people, ages 16–25 years, with a diagnosis of IA (JIA, ankylosing spondylitis [AS], psoriatic arthritis [PsA], or rheumatoid arthritis [RA]). Subject to meeting nationally agreed upon criteria, young people in England can access a range of treatments, including biologic therapies, without charge, from the National Health Service (NHS).

**Figure 1:**
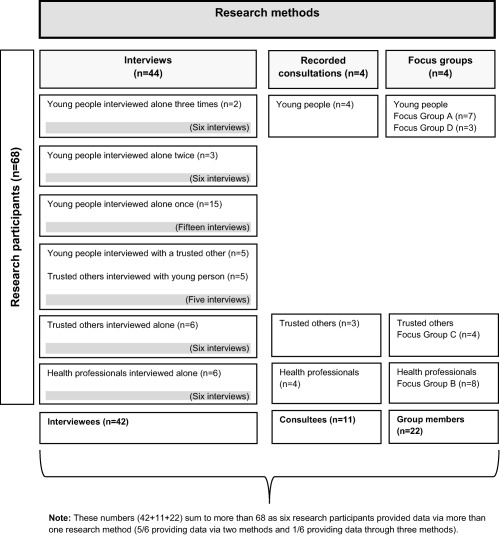
Map of research methods and research participants.

The study used multiple methods and sources, an approach termed methodological and data triangulation 12. Methods used included interviews (n = 44), audio‐recordings of consultations (n = 4), and focus groups (n=4). Research participants (n = 68) were young people (n = 37), trusted others (n = 15), and health professionals (n = 16). Figure 1 maps methods against participants. Participants were recruited via 3 NHS Hospital Trusts running adolescent, young adult, and/or adult rheumatology clinics. All participants consented verbally and in writing and the study had research ethics committee approval (Yorkshire & Humber, Leeds East). The research complied with the Declaration of Helsinki.

#### Interviews

Twenty‐five young people were interviewed, recruited purposively to ensure diversity (in demographic characteristics, diagnosis, and treatment history) and support exploration of emerging issues (see Table 1). The sample included 5 young people who at the first interview had not yet been offered a biologic agent, 5 who had recently been offered a biologic agent, and 15 taking (or who until recently had taken) a biologic agent. Where treatment status changed, we sought to reinterview (3 young people were interviewed twice, and 2 others were interviewed 3 times). Young people were recruited with the help of the direct care team, which identified patients with specified characteristics and made initial approaches (giving young people written information and seeking permission to pass on their contact details).

**Table 1:** Characteristics of young people interviewed (n = 25)

Characteristic	No.
Diagnosis	
Juvenile idiopathic arthritis	15
Ankylosing spondylitis	7
Psoriatic arthritis	2
Rheumatoid arthritis	1
Female	15
Mean (range) age, years	20 (16–25)
Ethnicity	
White British	24
Mixed	1
Mean (range) disease duration, years	9 (<1 to >20)
Type of medication taken at time of final interview	
Biologic agents	19
Disease‐modifying antirheumatic drugs	8
Oral steroids	1
Nonsteroidal antiinflammatory drugs	8
No medication	3
Rheumatology service accessed	
Adult clinic	10
Young adult clinic	8
Adolescent clinic	7

Eleven trusted others also took part in an interview (8 mothers, 1 father, 1 grandmother, and 1 partner). Trusted others were identified by the participating young people. In 5 instances they were interviewed with the young person. Trusted others who did not accompany young people to interviews were approached through them. Young people gave trusted others written information on the study and asked for permission to pass on their contact details. Most agreed to participate; those declining included a close friend and a partner.

Six health professionals were interviewed, including nursing and medical staff from all 3 types of clinic and trusts, recruited to provide a range of perspectives. Interviewees were proposed by the research team and approached directly by the researcher with written information.

Interviews lasted 40–120 minutes. Most were conducted face‐to‐face, at a location of the interviewee's choice. All interviews were semistructured, using schedules informed by the team's experience, a review of the literature, and the emerging analysis. These addressed a set of core topics (e.g., the decision‐making process, information exchange, views on risks and benefits) but differed in detail and emphasis to reflect individual circumstances (e.g., young people's treatment history and the specifics of professionals’ roles).

#### Recorded consultations

Four consultations were recorded involving 11 participants (different combinations of young people, trusted others, and health professionals). We hoped to compile a larger body of recordings, but negotiating and arranging these proved challenging. The recordings should be considered a convenience sample. However, they include a short first conversation about biologic therapies and a lengthier counseling session. They provide a detailed record of how biologic therapies are explained and the questions and concerns that arise.

#### Focus groups

Four focus groups were convened at the end of the project to explore the face validity of findings. Focus group A comprised 7 young people (5 female, 2 male; ages 16–20 years; 5 with JIA, 2 with other diagnoses) and focus group D comprised 3 young people (all male; ages 17–22 years; 2 with JIA, 1 with AS). Focus group B comprised 8 health professionals with interests in adolescent rheumatology. Focus group C comprised 4 trusted others (3 mothers, 1 grandmother). Recruitment to the focus groups was purposive (invitations being extended to people with and without prior involvement in the research). The groups provided a forum in which participants could comment on the intelligibility, credibility, and significance of findings and invite further reflection on the analyses.

#### Data analysis

Interviews, focus groups, and consultations were audio‐recorded and transcribed verbatim for analysis. Transcripts were analyzed by an author (RIH) using techniques foundational to grounded theory (open and focused coding, constant comparison, memoing, mapping) to identify patterns and relationships 13, 14. Data were analyzed within and across samples. The principal investigator (TR) provided a check by analyzing data segments (selected transcripts or data on a particular theme). Analyses were tested further with other researchers in biweekly data clinics and at biannual team and steering group meetings. Analysis ran alongside and informed sampling.

## RESULTS

We report here on a series of related themes in our data, all underpinned by the concept of “being normal.” This was a high priority for young people in our study, informing the processes of making and evaluating decisions. Put simply, young people aspired to live a normal life (theme 1); treatment was perceived as both an opportunity for and a threat to this (theme 2). Powerful emotions were a context for and a consequence of treatment decisions (theme 3). Information relevant to such decisions was acquired from different sources (theme 4), which did not always align. Decisions were considered provisional (theme 5) and were reviewed against experience. The focus on short‐term outcomes (theme 6) was a concern to trusted others and professionals.

#### Theme 1. Aspiring to live a normal life

Many of the young people interviewed talked of how they aspired to live what they perceived as a normal life, or as one put it, “to get back to … a normal way of life … not really too different from anybody else's” (male, age 21, AS). Not all the young people taking part in the focus groups connected with the term “normal,” but they accepted that its components had resonance: “Living a normal life is a priority? I would agree with [that], but don't like the word ‘normal”’ (male, age 24, AS).

“Normal” is a complex, multifaceted, situational, and dynamic concept concerning not only bodily function and experience, but also mental well‐being and performance of social and vocational roles. One professional explained: “They want to be able to get up in the morning and just be able to move. They want to go to work. They want to stay at college. They want to complete their university degree. They want to travel. They want to do normal things” (health professional).

Young people constructed their ideas of normal through reference to alternate selves (pre‐diagnosis or on a good day) and to others. They engaged in processes of implicit and explicit comparison, with unknown, idealized others (young people in the abstract) and known others (e.g., siblings and friends). These known others seemed a particularly important reference point. Young people wanted to feel, look, think, and act like them. They largely wanted their peers to think they were like them, and to treat them accordingly. For some young people this hope was realized, but for others, the sense of being different (a word commonly juxtaposed with normal) ran deep.

#### Theme 2. Seeing treatment as an opportunity and as a threat

In the context of aspiring to live a so‐called normal life, treatment appeared to be both an opportunity and a threat. Most young people confronted with treatment decisions were keen to experience relief from symptoms. A change in treatment might reduce pain, improve mobility, and get life “back to normal.” Effective management of their condition enabled young people to make plans for the future. This included taking steps into education or employment, or toward independent living: “I'm thinking of going back to college in September. Just because I feel like everything is being managed now. I feel I've a better chance of going to college and actually being able to stay there” (female, age 24, PsA).

However, those experiencing side effects from prior treatments were acutely aware of the potential for less positive outcomes, presenting threats to normal life. Some young people (and trusted others) described how steroids had caused changes in face shape and weight. Many talked of how methotrexate had triggered nausea, vomiting, and vocational underperformance: “Every Monday, you could guarantee that I wasn't at school … 'cause I was still sick from the medicine” (female, age 17, JIA). Others explained how increased susceptibility to infections had diminished their general sense of well‐being and disrupted their lives.

Even where a drug proved effective in controlling inflammatory processes, relieving associated symptoms, and had minimal side effects, it could be highly intrusive. Several young people explained how their plans and routines were dictated by their treatment schedule. The loss of freedom to engage in activities taken for granted by their peers was lamented, compounding a sense of being different: “All my friends are going out, going clubbing, going camping. And I always have to think about my medication and that first, before I even think about anything else” (female, age 22, JIA).

#### Theme 3. Experiencing powerful emotions

Many young people revealed anxieties about aspects of treatment. Methotrexate cast a long shadow over some young lives: “The look of it, the smell of it, the very thought of it made me shake” (male, age 17, JIA). The psychosocial sequelae of treatment included familial tensions, isolation, and bullying. Decisions about treatment changes were consequently highly charged: seemingly small adjustments to dosage, routines, or mode of administration could unleash strong emotions. Escalation of treatment forced young people to confront their condition, challenging efforts to perceive and present themselves as normal. Such decisions also brought them face‐to‐face with the uncertainties of the future. Most recognized that no treatment was guaranteed to work, and that their options were becoming more limited. Young people tried to maintain an optimistic outlook, but past disappointments bred caution: “The methotrexate worked for a little bit and then it stopped working … to put all your hopes on (adalimumab) seems a bit … I can't really do that” (female, age 24, PsA).

#### Theme 4. Acquiring information from different sources

In evaluating treatment changes, young people drew on information from various sources. They gained much of their knowledge, particularly about biologic agents, from health professionals delivering their care. However, this was supplemented by information from family or friends with relevant experience or expertise, research done by and for young people, and, critically, direct personal experience.

Evidence from personal experience played a powerful role in shaping young people's understanding of their condition and the treatments used to manage it. Prior treatments, including for other conditions, provided important reference points: “My attitudes toward potential outcomes with things like this are colored by a lot of the treatments I've received as a child” (male, age 22, AS). Often experiential evidence aligned with clinical measures, but not always: “Sometimes I feel like I'm just a blood results number. They, they're looking at my blood results—yes, my blood result may be sky high, but I feel perfectly fine” (female, age 20, JIA). Conflicting evidence could lead to frustration (on all sides) and fueled a sense of uncertainty.

#### Theme 5. Making provisional decisions

Young people emphasized the uncertainty associated with a new treatment, with respect to both its effects and challenges: “Nobody knows which one's best (etanercept or adalimumab) … it's a bit of a shot in the dark” (male, age 25, AS). New treatments were judged against other (past or present) treatments and, less frequently, the uncontrolled condition. Often the push of a certain (and intolerable) past or present treatment outweighed the pull of a future treatment: “I was willing to try it (etanercept) because I hated methotrexate” (female, age 16, JIA).

In the face of uncertainty, treatment decisions were considered provisional and open to review. Having gathered information on treatment administration, associated restrictions, anticipated effects, and potential side effects, the approach most often adopted was “try it and see.” Ultimately the test was whether treatment made life “easier … rather than harder” (female, age 22, JIA). This required more than just an improvement in symptoms; also important were side effects that were at most “annoying,” minimal restrictions, and a relatively simple and stable regimen. Collectively, these things acted to increase or diminish the sense of living a “normal” life. Where life did not feel more normal, commitment to treatment waned. Commitment could be reinforced by experiential evidence acquired from suspension of treatment: “When I came off medication, and I'd flare a bit or something, I realized how much difference it's doing … that has made me understand how they are, how it is doing me good … although sometimes, some days, it feels like it's making me worse” (male, age 16, JIA). Both young people and trusted others described trialing withdrawal of treatment; other reasons for breaks included infection, surgery, travel, conception, and oversight.

Where the impact of treatment was rapid and clear cut, as was often the case with biologic agents, the “try it and see” approach was unproblematic. However, with drugs such as methotrexate, where benefits took longer to emerge and initial side effects could be onerous, decisions were often reconsidered. Some young people (or trusted others) consulted care teams about alternatives. However, not all were aware that there were alternatives, and could wait until their next appointment to discuss them. In such circumstances the potential for unilateral discontinuation or partial nonadherence to treatment was high, with care teams becoming fully aware of young people's difficulties only when a crisis point was reached.

#### Theme 6. Focusing on the short term

Trusted others and health professionals perceived young people as focused on short‐term outcomes: “My worry is always years ahead, where [my daughter] wouldn't worry about [the future] at all. That will not even feature in her, in her mind” (mother of female, age 17, JIA). They expressed concern that young people might not take on the longer‐term risks of, on the one hand, taking treatment and, on the other, not taking treatment: “You're giving [treatment] to them to help them live a normal life. But there's much more to it than that. You give it to them … to stop things happening that they couldn't even begin to imagine” (health professional).

Short‐term concerns are more prominent in the data from young people than longer‐term concerns. The minority vocalizing longer‐term concerns had more complex treatment histories or a history of cancer in the family. More commonly, young people noted the long‐term risks briefly, framing them as low probability and easily resolved. A common assertion was that doctors would not propose treatment options if the risks were unreasonable: “The view I take is that if it's been offered to me, it's safe” (male, age 25, AS). However, a small minority reflected that when beginning certain treatments, they had not appreciated that they might be taking them long term.

Lack of attention to the long‐term risks was for some young people an active choice. Many stressed how difficult it was to think beyond the immediate future when life was made so challenging by their condition and/or treatment. They felt bound to accept the long‐term uncertainties or, as one interviewee put it, the “what ifs,” in order to get on with life in the short term. They acknowledged, but tried not to dwell upon, the future effects of treatment: “If something bad happens, I'll cross that bridge when I come to it. For now, it's just keeping me normal. I know that sounds a bit reckless, and I don't mean it like that, but like, I can't worry about what, you know, what would happen” (female, age 24, PsA).

## DISCUSSION

Despite the growing interest in patient preferences, to date little attention has been given to those of young people with IA. Lipstein et al 15 describe treatment decision‐making by adolescents with chronic illness generally as an understudied area. Their work, which includes young people with JIA, focuses on early adolescence (ages 10–15 years). Our research on older adolescence and early adulthood (ages 16–25 years) extends and complements that. It provides new insights on the perspectives of an important but neglected patient subgroup, and on how young people's experiences are understood by those around them (trusted others and health professionals).

Our study shows clearly how young people with arthritis, similarly to those with other chronic conditions 16, 17 aspire to live so‐called normal lives, but that both IA and its treatment present challenges to this. We recognize that young people are not alone in valuing treatments promoting “normal” life: studies of older adults with arthritis have drawn similar conclusions 3, 18. However, our research suggests that the features of normal life in adolescence and early adulthood are distinct, as are the consequences of not being normal or not being perceived as normal. It shows how participation in developmental and peer‐group activities acts as a litmus test for normality, and constraints upon participation may negatively affect well‐being, careers, and relationships. Williams et al note how a socially derived concept of normality becomes more prominent in adolescence 17. In illuminating how this informs young people's treatment preferences, our work reinforces and extends the findings of other research on chronic illness in adolescence 19, 20, 21.

Treatment developments in rheumatology have challenged thinking about the disruptive nature of chronic illness 22, 23. New medications have been framed as restorative by some authors 24. However, treatments such as methotrexate are widely recognized as having unpleasant side effects 25. Beyond rheumatology, increasing attention is being given to the practical and cognitive burdens that treatment regimens place on patients 26. Our study reflects this complex picture, showing how for young people with arthritis, treatment is a double‐edged sword, and adding to evidence suggesting that clinical outcome measures do not capture all that matters to patients 3, 27, 28.

Our research highlights the preferences of a diverse group of young patients, but is no substitute for careful exploration of the concerns of individuals. Other authors have stressed the importance of dialogue with older patients before and after the initiation of treatment 5. This is no less the case for young people; indeed they may need more active encouragement, and a wider range of channels, to raise concerns and articulate difficulties. A central message from our work is that it must be made absolutely clear to young people that concerns about the impact of treatment on, among other things, appearance, relationships, or education, are valid things to raise. A recent study has shown that bidirectional sharing of information in pediatric consultations about biologic agents is uncommon 29. Clearly, a more collaborative approach to considering, constructing, and sharing preferences is needed. Models such as shared decision‐making and collaborative deliberation offer relevant processes and guidance 30, 31.

A key concern of trusted others and health professionals was young people's perceived focus on the short term. Care must be taken not to discount prioritization of short‐term gains as a reflection of the adolescent brain. While neuroscience offers plausible explanations for such a short‐term perspective, social context has been shown as important 8. Moreover, older adults with arthritis have been found to have a similar focus 2, 18, 28. This is not to dispute the importance of making sure that young people are well informed about risk‐benefit tradeoffs or longer‐term consequences of not taking clinically optimal medication (or not taking medication the optimal way) 15. Young people have themselves reported wanting transitional care programs to cover rationales for treatment, side effects, and delays in observation of benefit 32. We believe that there is a strong case for periodically revisiting the long‐term risks and benefits of treatment decisions (to take or not), both as a prompt to young people to air concerns and to check understanding.

Our research has both strengths and weaknesses. The nature of qualitative work is that it is in‐depth but small scale, with a consequent strength being the richness of data and a weakness being the low number of cases. However, the body of data compiled during this study is relatively substantial and the number of participants (n = 68) relatively large. Conducting serial and triangulated interviews enabled us to confirm stories and capture evolving perspectives 33. Recording consultations enabled us to compare what people do against what they say they do in these contexts. The use of focus groups to explore the face validity of our findings (also referred to as member validation) strengthens our confidence in their credibility 34.

In conclusion, this study provides important insights into young people's circumstances and preferences and how these may inform treatment decisions. It challenges assumptions, implicit in much of the previous research, that studies of the typical (i.e., older) patient provide a basis for understanding young people's priorities. Our data reveal age as an important factor in the evaluation of treatment options, underpinning perceptions of “normal” life, issues achieving and maintaining this, and the consequences of not doing so. But to find out what matters to any particular young person, we must ask questions, set aside our assumptions, and listen to what he or she has to say. Only through understanding young people's values, preferences, and concerns can a sustainable balance between disease control and treatment burden be achieved.

## AUTHOR CONTRIBUTIONS

All authors were involved in drafting the article or revising it critically for important intellectual content, and all authors approved the final version to be submitted for publication. Ms Hart had full access to all of the data in the study and takes responsibility for the integrity of the data and the accuracy of the data analysis.

**Study conception and design.** McDonagh, Thompson, Foster, Kay, Rapley.

**Acquisition of data.** Hart, McDonagh, Thompson, Foster, Kay, Myers.

**Analysis and interpretation of data.** Hart, Rapley.
